# Supplementary material for: Differences in Bacterial Communities of Retail Raw Pork in Different Market Types in Hangzhou, China
Source: Foods. 2023 Sep 7;12(18):3357. doi: 10.3390/foods12183357 (PMC10529276; doi:10.3390/foods12183357)
Supplement: Supplementary file 1 [file foods-12-03357-s001.zip › foods-2582001-supplementary.pdf]

Table S1. Diversity indices for 84 pork samples used in this study.

	Observed OTUs	Shannon	Simpson	Chao1	Goods_coverage
FA†	623±102	6.32±0.61	0.94±0.03	640.85±104.07	0.9987±0.0004
FB	672±81	6.75±0.40	0.97±0.01	693.00±87.12	0.9986±0.0004
FC	619±53	6.75±0.33	0.97±0.01	635.57±54.14	0.9988±0.0003
FD	441±87	5.68±1.07	0.92±0.05	457.35±86.51	0.9990±0.0003
FE	597±111	6.47±0.48	0.96±0.02	609.54±112.33	0.9990±0.0003
USA	342±54	4.37±0.24	0.84±0.03	354.56±61.12	0.9992±0.0003
USB	443±13	5.43±0.11	0.91±0.02	455.19±16.23	0.9991±0.0002
USC	428±133	4.34±0.28	0.76±0.04	450.42±139.47	0.9986±0.0006
USD	396±42	5.15±0.32	0.89±0.03	416.80±57.28	0.9989±0.0005
USE	448±72	5.67±0.53	0.92±0.03	462.63±80.13	0.9989±0.0006
OSA	254±132	4.79±0.96	0.89±0.05	259.43±134.33	0.9996±0.0002
OSB	335±175	5.40±1.03	0.93±0.02	337.56±177.06	0.9997±0.0002
OSC	364±66	5.24±0.12	0.92±0.01	369.08±69.99	0.9995±0.0003
OSD	245±24	4.37±0.27	0.87±0.04	251.69±24.84	0.9996±0.0001
OSE	252±39	4.90±0.24	0.91±0.01	256.26±40.19	0.9996±0.0002
